# PNPLA3 has retinyl-palmitate lipase activity in human hepatic stellate cells

**DOI:** 10.1093/hmg/ddu121

**Published:** 2014-03-25

**Authors:** Carlo Pirazzi, Luca Valenti, Benedetta Maria Motta, Piero Pingitore, Kristina Hedfalk, Rosellina Margherita Mancina, Maria Antonella Burza, Cesare Indiveri, Yvelise Ferro, Tiziana Montalcini, Cristina Maglio, Paola Dongiovanni, Silvia Fargion, Raffaela Rametta, Arturo Pujia, Linda Andersson, Saswati Ghosal, Malin Levin, Olov Wiklund, Michelina Iacovino, Jan Borén, Stefano Romeo

**Affiliations:** 1 Department of Molecular and Clinical Medicine, Institute of Medicine, Sahlgrenska Center for Cardiovascular and Metabolic Research, Wallenberg Laboratory and; 2 Department of Chemistry and Molecular Biology, University of Gothenburg, Gothenburg, Sweden; 3 Department of Pathophysiology and Transplantation, University of Milan, Milan, Italy; 4 Department BEST (Biologia, Ecologia, Scienze Della Terra), Unit of Biochemistry and Molecular Biotechnology, University of Calabria, Arcavacata di Rende, Italy; 5 Department of Medical and Surgical Sciences, Clinical Nutrition Unit, University Magna Graecia of Catanzaro, Catanzaro, Italy; 6 Department of Pediatrics, LA Biomedical Research Institute at Harbor-UCLA, 1124 W. Carson Street, HH1, Torrance, CA 90502, USA

## Abstract

Retinoids are micronutrients that are stored as retinyl esters in the retina and hepatic stellate cells (HSCs). HSCs are key players in fibrogenesis in chronic liver diseases. The enzyme responsible for hydrolysis and release of retinyl esters from HSCs is unknown and the relationship between retinoid metabolism and liver disease remains unclear. We hypothesize that the patatin-like phospholipase domain-containing 3 (PNPLA3) protein is involved in retinol metabolism in HSCs. We tested our hypothesis both in primary human HSCs and in a human cohort of subjects with non-alcoholic fatty liver disease (*N* = 146). Here we show that PNPLA3 is highly expressed in human HSCs. Its expression is regulated by retinol availability and insulin, and increased PNPLA3 expression results in reduced lipid droplet content. PNPLA3 promotes extracellular release of retinol from HSCs in response to insulin. We also show that purified wild-type PNPLA3 hydrolyzes retinyl palmitate into retinol and palmitic acid. Conversely, this enzymatic activity is markedly reduced with purified PNPLA3 148M, a common mutation robustly associated with liver fibrosis and hepatocellular carcinoma development. We also find the PNPLA3 I148M genotype to be an independent (*P* = 0.009 in a multivariate analysis) determinant of circulating retinol-binding protein 4, a reliable proxy for retinol levels in humans. This study identifies PNPLA3 as a lipase responsible for retinyl-palmitate hydrolysis in HSCs in humans. Importantly, this indicates a potential novel link between HSCs, retinoid metabolism and PNPLA3 in determining the susceptibility to chronic liver disease.

## INTRODUCTION

Retinoids are liposoluble micronutrients that are highly enriched in the retina and stellate cells (1,2). Besides playing a major physiological role in vision, they are also involved in the regulation of cell proliferation and differentiation (3,4). In addition, retinoids are used in the treatment of malignancies (4,5) and have been shown to prevent hepatocellular carcinoma (6,7). In humans, retinoids are absorbed in the intestine and transported in the form of retinyl esters by chylomicrons to the liver (8). Retinyl esters are hydrolyzed to retinol in hepatocytes and then transferred to hepatic stellate cells (HSCs) and stored as retinyl palmitate in lipid droplets (9). When needed, the retinol stored in lipid droplets can be released to reach the specific sites where it exerts its physiological functions (10). In the blood stream, retinol is transported by the retinol-binding protein 4 (RBP4) (11), a protein belonging to the lipocalin family synthesized by hepatocytes (12). The plasma levels of RBP4 strongly correlate with plasma retinol levels (13). Furthermore, the RBP4 genetic locus is the major genetic determinant of plasma retinol levels (14).

In response to liver damage, HSCs are activated from a quiescent to a profibrotic state (15); importantly, this process is coupled to release of retinol (10). However, the lipases responsible for the release of retinol from HSCs in response to metabolic stimuli are not known, and the metabolic relevance of this event is still not understood (1,2).

Patatin-like phospholipase domain-containing 3 (PNPLA3) has a lipase activity against triglycerides in human and murine hepatoma cell lines (16,17). A naturally occurring sequence variation (rs738409) resulting in a isoleucine (I) to methionine (M) substitution at position 148 (I148M) (18) of the protein is the most robustly replicated genetic variant associated with liver damage (19), hepatic neutral fat retention (17) and progression to chronic liver disease (20–23). Despite the strong associations, the underlying mechanism remains unknown.

Here, we hypothesized that PNPLA3 is involved in retinol metabolism in HSCs. We examined the regulation of PNPLA3 in response to changes in retinol availability and metabolic needs, exemplified by insulin, in primary human HSCs (pHSCs). Furthermore, we investigated the effect of the I148M mutation on retinol metabolism in human HSCs and we tested for differences in circulating levels of RBP4 among individuals carrying different PNPLA3 I148M genotypes.

## RESULTS

### PNPLA3 is highly expressed in human HSCs

**Figure 1.**
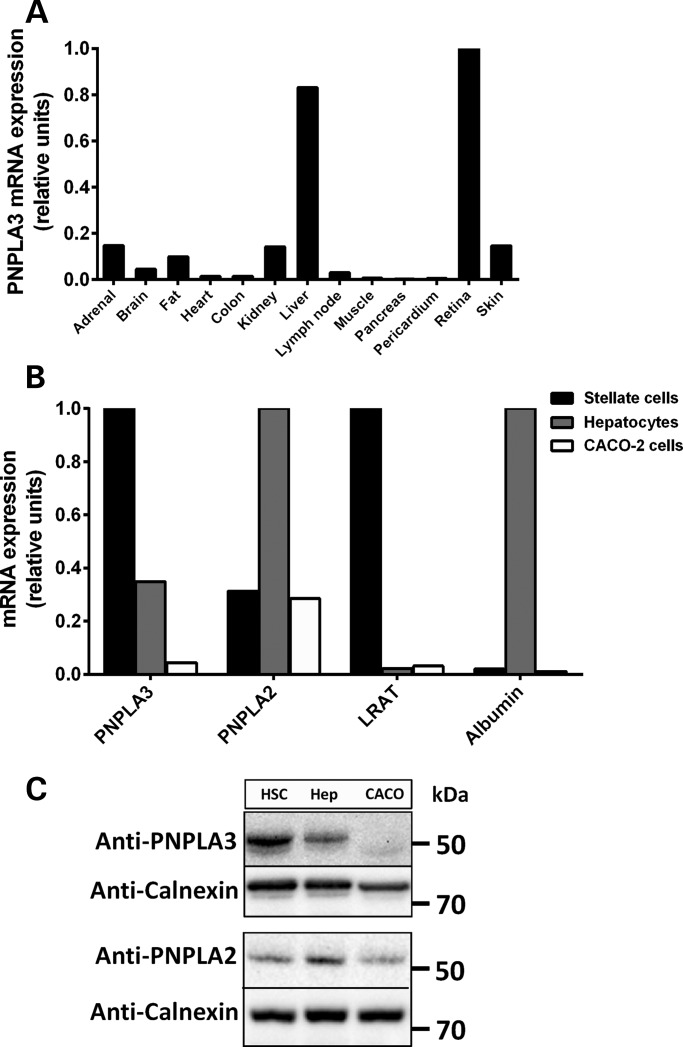
PNPLA3 is highly expressed in human retina, hepatocytes and HSCs. (**A**) PNPLA3 mRNA expression in human tissues assessed by qPCR. The tissue with the highest CT value was assigned the value of 1. (**B**) PNPLA3 mRNA expression in pHSCs, primary human hepatocytes (positive control) and human colon carcinoma (CACO-2) cells (negative control). Lecithin retinol acyl transferase (LRAT) and albumin were used as references for pHSCs and hepatocytes, respectively. (**C**) Immunoblot showing PNPLA3 and PNPLA2 protein expression in pHSCs, primary human hepatocytes (Hep) and CACO-2 cells. Calnexin was used as a loading control.

We performed an extensive analysis of *PNPLA3* mRNA expression in human tissues (Supplementary Material) and found the highest expression in the retina and liver (Fig. 1A and Supplementary Material, Fig. S1). We next evaluated the relative expression levels of PNPLA3 within the liver in primary human hepatocytes and HSCs. PNPLA3 mRNA and protein expression levels were high in pHSCs compared with hepatocytes; the levels of PNPLA3 were also high in comparison with PNPLA2, the major triglyceride esterase in hepatocytes and adipocytes (Fig. 1B and C). Finally, we examined the protein levels of three lipases (24) that have been proposed as candidate for the retinyl-esterase activity. The protein levels of all three candidate lipases were almost undetectable in LX-2, an immortalized human HSC line (Supplementary Material, Fig. S2).

### PNPLA3 is regulated by retinol availability in human HSCs

**Figure 2.**
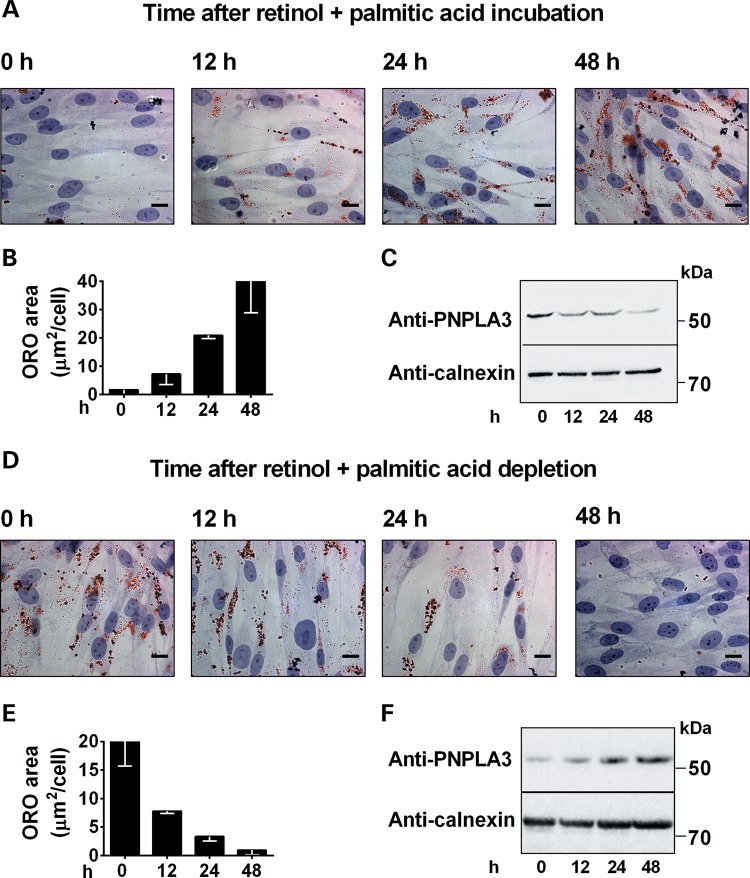
Intracellular lipid accumulation modulates PNPLA3 expression in pHSCs. (**A**) Lipid droplet content visualized by ORO-staining in pHSCs incubated with retinol and palmitic acid for the indicated times. (**B**) ORO-stained area quantified by Biopix. (**C**) Immunoblot showing PNPLA3 expression in pHSCs under conditions described in (A). (**D**) Lipid droplet content visualized by ORO staining in pHSCs incubated with retinol and palmitic acid for 48 h and then in medium without retinol-palmitic acid for the indicated times. (**E**) ORO-stained area quantified by BioPix. (**F**) Immunoblot showing PNPLA3 expression in pHSCs under conditions described in (D). Scale bars: 10 µm.

To characterize the regulation of PNPLA3 expression in HSCs in response to retinol availability, we incubated pHSCs with or without retinol and palmitic acid for up to 48 h. In the presence of retinol and palmitic acid, we observed a time-dependent increase in lipid droplet accumulation paralleled by a downregulation of PNPLA3 (Fig. 2A–C). Depletion of retinol and palmitic acid resulted in a time-dependent reduction in lipid droplet accumulation and an increase of PNPLA3 expression (Fig. 2D–F). Identical results were obtained in LX-2 cells (data not shown).

### Insulin-mediated upregulation of PNPLA3 promotes a reduction of lipid droplet content in human HSCs

**Figure 3.**
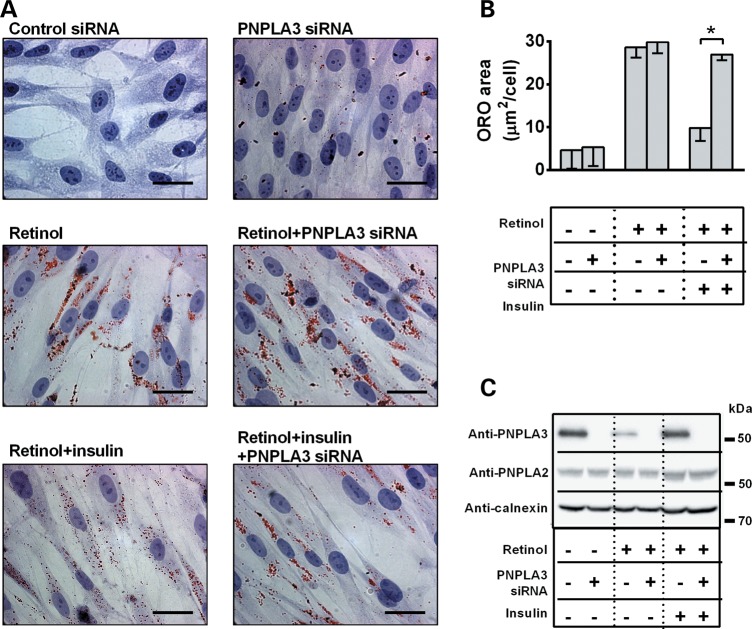
Insulin-mediated PNPLA3 upregulation reduces lipid droplet content in pHSCs. (**A**) Lipid droplet content visualized by ORO staining in pHSCs incubated w/o retinol-palmitic acid for 48 h, transfected with PNPLA3 or control siRNA and incubated w/o insulin for further 48 h. Scale bars: 10 µm. (**B**) ORO-stained area quantified by BioPix. *P < 0.05. (**C**) Immunoblot showing PNPLA3 and PNPLA2 expression in pHSCs under conditions described in (A).

PNPLA3 is upregulated by insulin through sterol regulatory element-binding protein 1C in hepatocytes (25), and here we observed higher protein levels of PNPLA3 in pHSCs after incubation with insulin (Supplementary Material, Fig. S3). We also observed a striking reduction in lipid droplet content associated with PNPLA3 upregulation after insulin incubation (Fig. 3A–C). This effect was abolished after PNPLA3 knockdown by siRNA (Fig. 3A–C), thus suggesting a direct role of PNPLA3 in insulin-dependent regulation of lipid droplet content in pHSCs. Identical results were obtained in LX-2 cells (Supplementary Material, Fig. S4).

### PNPLA3 promotes extracellular release of retinol from human HSCs but not triglyceride hydrolysis

**Figure 4.**
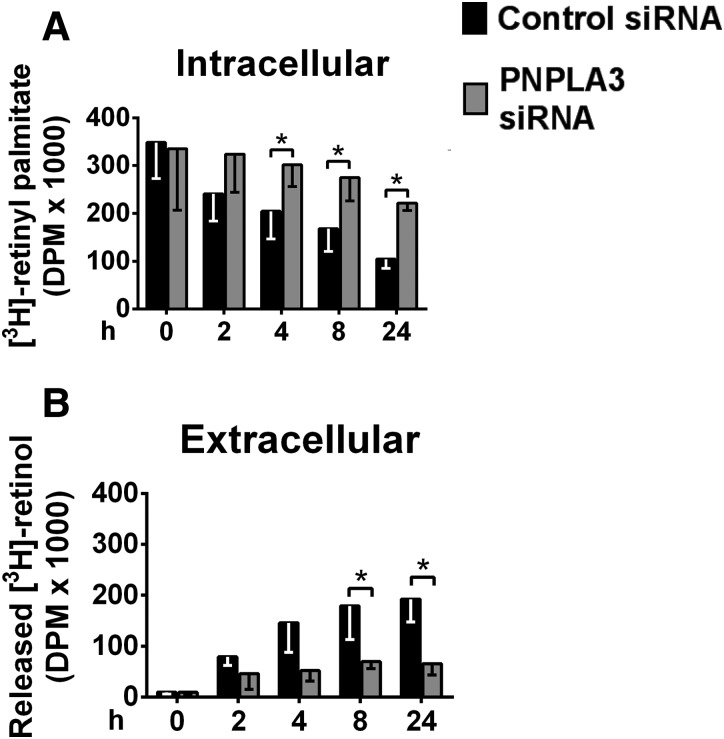
PNPLA3 silencing reduces retinol release in LX-2 cells. (**A**) Intracellular [^3^H]-retinyl palmitate and (**B**) extracellular [^3^H] retinol from LX-2 cells transfected with PNPLA3 or control siRNA, incubated with [^3^H]-retinol-palmitic acid and chased in cold medium for the indicated times. *P < 0.05.

To establish the specific role of PNPLA3 in retinyl-palmitate hydrolysis, we investigated the effect of PNPLA3 silencing on the intracellular content of retinyl palmitate and the extracellular release of retinol from differentiated LX-2 cells upon retinol depletion from the culture medium. In the presence of control siRNA, the intracellular content of retinyl palmitate decreased over time in parallel with an increased release of retinol (Fig. 4A and B). By contrast, treatment with PNPLA3 siRNA reduced both the intracellular depletion of retinyl palmitate and the extracellular release of retinol (Fig. 4A and B). These data suggest that PNPLA3 is involved in the hydrolysis of retinyl palmitate to retinol and palmitic acid in HSCs.

To test PNPLA3 lipase activity on glycerolipids in stellate cells, we examined the impact of PNPLA3 I148M mutation on triglyceride storage in LX. Cells overexpressing the 148I wild type or 148M mutant PNPLA3 were incubated with radiolabeled palmitic acid and intracellular triglycerides were examined after lipid fractionation. No differences in the intracellular triglyceride storage were observed between cells overexpressing the PNPLA3 wild type or the mutant form or transfected with the empty vector (Supplementary Material, Fig. S5), suggesting the absence of PNPLA3 triglyceride hydrolase activity in stellate cells.

### Human PNPLA3 I148M is a loss-of-function mutation in HSCs

**Figure 5.**
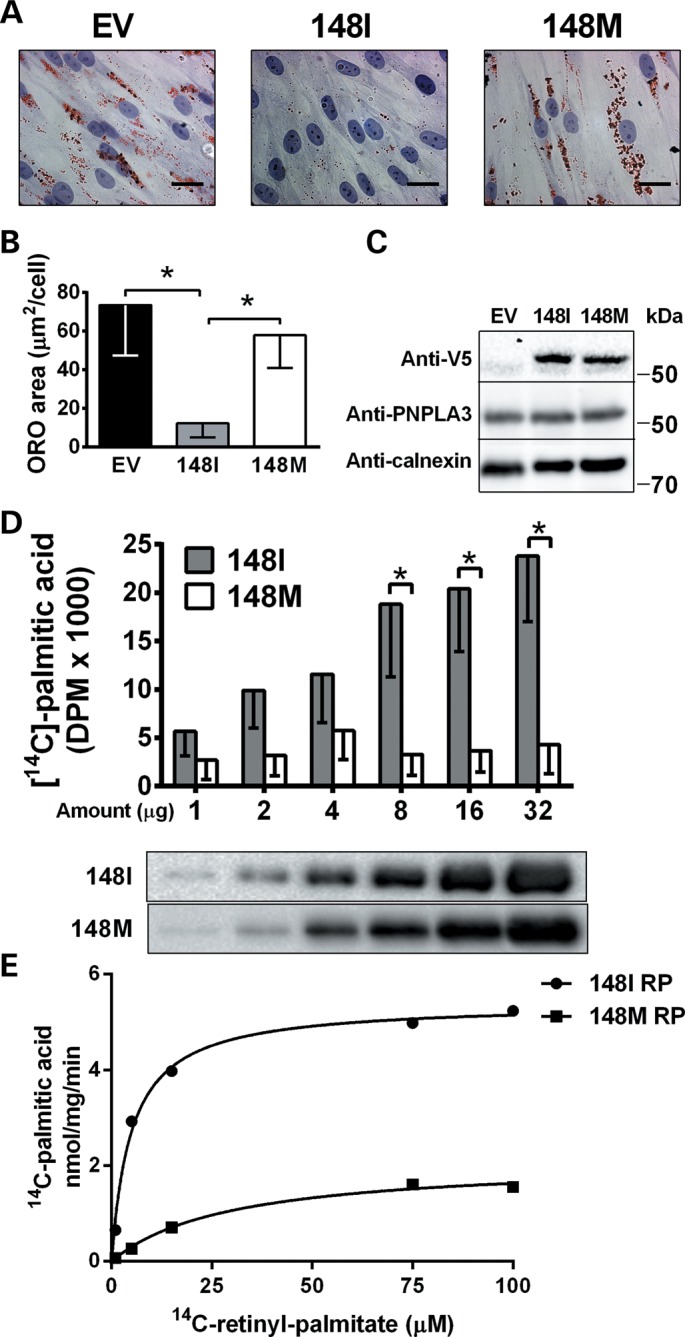
Overexpression of wild type but not mutant PNPLA3 reduces lipid droplet content in pHSCs and the mutation induces retinyl-hydrolase activity loss of function. (**A**) Lipid droplets visualized by ORO-staining in pHSCs overexpressing V5-tagged 148I or 148M PNPLA3 and incubated with retinol-palmitic acid for 48 h. Empty vector (EV) was used as negative control. (**B**) ORO-stained area quantified by BioPix. (**C**) Immunoblot showing transfection efficiency. (**D**) [^14^C]-palmitate production after incubation of the indicated amounts of purified human 148I and 148M PNPLA3 with retinyl [^14^C] palmitate for 15 min. (**E**) PNPLA3 148I and 148M purified proteins were incubated with increasing concentrations of radiolabeled retinyl palmitate. Released palmitic acid was measured by scintillation counting. Values were fitted to Michaelis–Menten kinetics curves to determine *V*_max_ and *K*_m_ values.

To further test the lipase activity and the effect of the I148M mutation, we overexpressed PNPLA3 148I wild type and the 148M mutant form in HSCs pretreated with retinol (Supplementary Material). Acute overexpression of PNPLA3 148I led to a striking reduction in lipid droplet content, whereas acute overexpression of PNPLA3 148M had no effect (Fig. 5A–C). Similar results were obtained in LX-2 cells (Supplementary Material, Fig. S6). These data indicate that the PNPLA3 148M is a loss-of-function mutation in terms of regulation of lipid droplet metabolism in human HSCs.

To assess the role of PNPLA3 in HSCs on the intracellular triglyceride content of hepatocytes, we performed a co-culture experiment. LX-2 and HEPG2 cells were co-cultured in a transwell plate. LX-2 cells were transiently transfected with PNPLA3 148I, 148M or the empty vector and the intracellular triglyceride content of hepatocytes was assessed by lipid fractionation after incubation with palmitic acid and radiolabeled glycerol. We observed no differences in intracellular hepatocyte triglycerides after co-culture with LX-cells overexpressing PNPLA3148I or 148M (Supplementary Material, Fig. S7). These data suggest that the triglyceride retention in hepatocyte is independent of retinol release by stellate cells.

### PNPLA3 148I but not 148M has retinyl esterase activity *in vitro*

To verify whether retinyl palmitate is a direct enzymatic substrate of PNPLA3, we purified the PNPLA3 148I wild type and the 148M mutant after recombinant production in a well-established eukaryotic host system (*Pichia pastoris*) (26,27). We incubated the purified proteins with radiolabeled retinyl [^14^C] palmitate and observed a concentration-dependent release of palmitic acid with PNPLA3 148I but not with the 148M protein (Fig. 5D). When we calculated the *K*_m_ and *V*_max_ by incubating the purified proteins with increasing concentrations of substrate, we observed a *V*_max_ reduction from 5.4 to 2.1 nmol/mg/min and a *K*_m_ increase from 4.9 to 29.7 μm in the 148M mutant protein compared with the 148I wild type (Fig. 5E). Thus, we confirmed that PNPLA3 148I has retinyl-palmitate lipase activity and that the I148M substitution results in loss of function.

### PNPLA3 has no retinyl-esterase activity in hepatocytes

To test if PNPLA3 in hepatocytes has a retinyl-esterase activity, we incubated cell lysates of rat hepatocytes (McA-RH7777 cells) stably overexpressing the human PNPLA3 wild type or 148M mutant protein (17) with radiolabeled retinyl palmitate and measured the palmitic acid release. A retinyl-esterase activity was observed for both lysates with no differences between the wild-type and the mutant protein (Supplementary Material, Fig. S8).

### PNPLA3 148M homozygotes have lower circulating levels of RBP4

To test the hypothesis that PNPLA3 protein is involved in retinol metabolism in humans, we compared RBP4 levels between carriers of the 148I allele and 148M homozygotes in a cohort of 146 individuals with biopsy proven non-alcoholic fatty liver disease (see Table 1; Supplementary Material, Table S1 for characteristics). A 12% reduction in RBP4 was found in PNPLA3 148M homozygotes compared with 148I allele carriers (II + IM = 52 ± 13 μg/mL, MM = 46 ± 17 μg/mL, *P* = 0.010). In a multivariate analysis, homozygosity for the M allele remained an independent predictor of lower RBP4 levels (*P* = 0.009, Table 2) after adjustment for factors previously associated with RBP4 plasma levels (age, gender, non-alcoholic steatohepatitis, body mass index and type 2 diabetes/impaired fasting glucose). These data are consistent with the hypothesis that PNPLA3 is involved in retinol release from HSCs and that intracellular retention of retinol occurs in 148M homozygotes.

**Table 1.** RBP4 analysis cohort description

Cohort characteristic	
*N*	146
Men (%)	78
Age (years)	49 ± 12
BMI (kg/m^2^)	27 ± 3
Glucose (mg/dl)	98 ± 27
RBP4 (μg/ml)	51 ± 14
NASH (%)	45
Diabetes or IFG (%)	35
PNPLA3 I148M genotype	
II (%)	40
IM (%)	44
MM (%)	16

*N*, number; BMI, body mass index; RBP4, retinol-binding protein 4; NASH, non-alcoholic steatohepatitis; IFG, impaired fasting glucose; PNPLA3, patatin-like phospholipase domain-containing protein 3; II, homozygous for the PNPLA3 148I allele; IM, heterozygous; MM, homozygous for the PNPLA3 148M allele.

**Table 2.** Multivariate analysis of factors modulating circulating RBP4 levels

	*β*	*P*-value
NASH	0.067	0.529
BMI	0.202	0.063
Age	0.004	0.966
Gender	0.206	0.042
DM or IGF	−0.048	0.633
PNPLA3 genotype	−0.250	0.009

Data were analyzed using linear regression analysis under a recessive model and after adjusting for confounding factors. Values were log transformed before entering the model if not normally distributed.

RBP4, retinol-binding protein 4; NASH, non-alcoholic steatohepatitis; BMI, body mass index; DM, diabetes mellitus; IFG, impaired fasting glucose; PNPLA3, patatin-like phospholipase domain-containing protein 3.

## DISCUSSION

Here we showed that PNPLA3 was highly expressed in retina and liver, with higher expression compared to other candidate retinyl-ester lipases in stellate cells and higher expression in pHSCs than in hepatocytes. PNPLA3 expression in pHSCs was regulated by retinol availability and insulin and was inversely related to lipid droplet content. Importantly, we showed that human PNPLA3 had a retinyl-esterase activity *ex vivo* and *in vitro* and that the I148M mutation resulted in a loss of this enzymatic activity.

A previous study reported high expression of PNPLA3 in the liver and skin (25). We showed that PNPLA3 was highly expressed in the retina and liver but we also observed an appreciable amount of PNPLA3 mRNA in the skin, consistently with the previous study. It is not possible to compare the retina mRNA PNPLA3 levels between the two studies.

We observed that endogenous PNPLA3 in pHSCs was downregulated by retinol incubation and upregulated by retinol depletion. These observations are consistent with the concept that PNPLA3 expression is (1) repressed when retinol is plentiful, resulting in storage of retinyl palmitate in lipid droplets and (2) induced in response to retinol deficiency, resulting in hydrolysis of retinyl palmitate and extracellular release of retinol.

Insulin has been shown to induce PNPLA3 expression in hepatocytes (25) and to activate HSCs (28). We observed that insulin treatment-induced PNPLA3 upregulation in pHSCs. Moreover, the insulin-induced increase in PNPLA3 levels was associated with a reduction of intracellular lipid droplets. Both these responses were abolished after PNPLA3 silencing. Together with our observation that PNPLA3 overexpression promoted a reduction of lipid droplets, these data indicate that PNPLA3 plays a specific role in intracellular lipid droplet remodeling in response to insulin in HSCs. It is noteworthy that changes in intracellular retinyl-ester content have been associated with HSC modifications during chronic liver damage (10).

We observed a time-dependent decrease in intracellular retinyl ester and a parallel extracellular release of retinol when pHSCs were preincubated with retinol and palmitate and then chased in retinol-free medium. These responses were reduced after PNPLA3 knockdown, suggesting that PNPLA3 has retinyl-esterase activity. The residual intracellular esterase activity observed after PNPLA3 knockdown may be explained by the presence of other lipases in HSCs.

Given the robust genetic evidence showing an association of PNPLA3 148M with liver neutral fat content and chronic liver diseases (29), we next examined the effect of acute overexpression of PNPLA3 148M in pHSCs. In contrast to the dramatic response observed in cells transfected with wild-type PNPLA3 148I, there was no difference in lipid droplet content between HSCs transfected with PNPLA3 148M or with an empty vector as a control. These data are in line with our previous study in hepatocytes, showing that overexpression of human PNPLA3 148I but not 148M promotes intracellular triglyceride lipase activity (17).

We have shown a lipase activity of the purified wild-type protein on retinyl palmitate. In a previous study that examined the PNPLA3 enzymatic activity on various glycerolipids, we found a predominant lipase activity on triglycerides and diglycerides but not on monoglycerides, indicating relative substrate specificity (30). Furthermore, PNPLA3 wild type has an ∼12-fold higher enzymatic affinity (*K*_m_ 4.9 versus 61.1) and a 3-fold lower enzymatic activity (*V*_max_ 14.7 versus 5.4) for the retinyl palmitate as compared with triglycerides (30). The PNPLA3 mutant form has a comparable reduction in both activity and affinity for triglycerides and retinyl palmitate (30).

We also tested if PNPLA3 wild-type and mutant protein has a retinyl-esterase activity in hepatocytes. We observed an overall retinyl-esterase activity but no differences in cells stably overexpressing the human PNPLA3 148I wild-type and 148M mutant protein. This suggests that the overall hepatocyte retinyl-esterase activity depends on the previously identified esterases (2), and PNPLA3 may not be active as a retinyl esterase in the hepatocyte. Furthermore, we failed to observe differences in hepatocyte intracellular fat content after co-culture with LX-2 cells overexpressing PNPLA3 148I or 148M, suggesting that the triglyceride retention in hepatocyte is independent of retinol release.

In humans, retinol in circulation is predominantly bound to the RBP4 (11), and RBP4 levels are highly correlated (*R*^2^ = 0.8) with circulating retinol (13). We examined the RBP4 levels in a cohort of 146 individuals stratified by PNPLA3 genotypes. To test if individuals with the 148M mutations had lower RBP4 protein we chose to use a recessive model. This decision was based on our earlier study showing that PNPLA3 I148M is associated with hepatocellular carcinoma under a recessive model (31). Homozygotes for the 148M allele had lower RBP4 compared with carriers of the 148I allele. Given that circulating retinol levels under fasting conditions are a function of retinol mobilization from HSCs these data are consistent with a model in which the PNPLA3 148M protein induces an intracellular retention of retinol due to a loss of the retinyl-palmitate esterase activity.

HSCs are involved in fibrogenesis (32) and retinoids have an important role in regulating cell growth, differentiation, carcinogenesis (4) and in the regulation of lipid metabolism in the liver (33). The present study identifies PNPLA3 as a possible link between HSCs, retinoids and chronic liver disease.

In mouse liver, *Pnpla3* mRNA has been shown to be expressed in HSCs at lower levels than in hepatocytes (25). However, tissue-specific differences between mouse and human in PNPLA3 have already been shown (25,34).

We propose that PNPLA3 represents a retinol gatekeeper in HSCs promoting release of retinol in response to metabolic needs. Additional studies are warranted to test whether PNPLA3 is involved in the transdifferentiation of HSCs in response to liver damage and to understand the role played by the I148M PNPLA3 variant in HSCs in the susceptibility toward hepatic fibrogenesis and carcinogenesis.

In summary, we identify PNPLA3 as the first retinyl-palmitate lipase in HSCs and we also show that the 148M mutation leads to a loss of this function. The importance of our study resides in establishing a new role for PNPLA3 in retinoid metabolism in HSCs with possible important implications for liver fibrosis and hepatocellular carcinoma susceptibility.

## MATERIALS AND METHODS

### Cell culture

Primary human HSCs (pHSCs; ScienCell) were grown in SteCM medium (ScienCell) in T-75 flasks coated with poly-l-lysine. When confluent, cells were trypsinized (0.05% trypsin/0.53 mm EDTA) and seeded at a ratio of 1 : 3. Subsequent passages were performed every 6 days. Immortalized human HSCs (LX-2) were kindly provided by Professor Scott L. Friedman (Mount Sinai School of Medicine). LX-2 cells were grown in high glucose DMEM containing 10% fetal bovine serum in T-75 flasks. When confluent, LX-2 cells were passaged as for pHSCs. Primary fresh human hepatocytes were purchased from 3H Biomedical. Human hepatoma (HEPG2) cells (ATCC) were grown in high glucose DMEM containing 10% fetal bovine serum in T-75 flasks according to the manufacturer's instructions. Colon carcinoma (CACO-2) cells (ATCC) were grown in MEM with Earl's salts according to the manufacturer's instructions. Cells were seeded in 24-well plates with cover slips (for Oil Red O staining, see Supplementary Material) or in 6-well plates (for immunoblot and mRNA expression analyses, see Supplementary Material).

### Analysis of retinyl-palmitate metabolism

Growing medium was supplemented with 10 µm retinol, 300 µm palmitic acid and 0.6 µCi/ml of [11,12-^3^H]-retinol and vigorously mixed to aid esterification to retinyl palmitate. Cells were seeded in 6-well plates and transfected with PNPLA3 siRNA or control siRNA for 12 h (Supplementary Material). Cells were then incubated with radiolabelled medium for 36 h, and retinol release was chased in cold medium for 0, 2, 4, 8 and 24 h. Cells and media were harvested at each time point and lipids extracted in 10 ml of Folch solution [chloroform : methanol (2 : 1 v/v) plus 2 ml of acidified solution (0.2 m orthophosphoric acid)]. After vigorous mixing and centrifugation at 3000*g* for 15 min at 4°C, the upper water phase was discharged and the lower organic phase was dried under nitrogen stream. Lipids were resuspended in chloroform and separated on a Silica plate by two-phase thin-layer chromatography (first mobile phase: chloroform : methanol : H_2_O 65 : 25 : 4; second mobile phase petroleum ether : diethyl ether : acetic acid 60 : 40 : 1). Lipids were next stained with iodine vapor and bands corresponding to retinol (for media samples) and retinyl palmitate (for cell samples) were counted for tritium by scintillation counting.

### Retinyl-palmitate hydrolase activity assay

PNPLA3 protein was purified as previously described (30). Briefly, 148I and 148M PNPLA3 cDNA were cloned in a pPICZ-B vector and transformed into *P. pastoris* for large-scale protein production. Proteins were solubilized and purified from the membrane fraction by Ni-affinity chromatography and subsequently used for the retinyl-palmitate hydrolase activity assay.

Increasing amounts of purified 148I wild type and 148M mutant PNPLA3 protein were incubated with retinyl [^14^C] palmitate for 30 min at 37°C. The reaction was terminated by adding heptane : chloroform : methanol (1 : 1.25 : 1.41), mixed, and centrifuged at 3000*g* for 15 min. A 1-ml aliquot of upper (water) phase was taken and released [^14^C]-palmitic acid was measured by scintillation counting. GraphPad Prism 5 software was used for Michaelis–Menten curve fitting and *V*_max_ and *K*_m_ value calculation.

### Study cohort

PNPLA3 I148M genetic variant was genotyped in 146 individuals with biopsy proven non-alcoholic fatty liver disease diagnosed between January 2008 and January 2013 at Fondazione IRCCS Ca’ Granda Ospedale Maggiore Policlinico Milano, Università degli Studi di Milano, Milan, Italy. Other causes of liver disease were ruled out, including increased alcohol intake (>30/20 g/day for M/F), viral and autoimmune hepatitis, hereditary hemochromatosis and alpha1-antitrypsin deficiency. This cohort recruitment has been previously been described (35), and this analysis includes a total of 146 individuals. Genotyping was performed as previously described (21). The study protocol was conformed to the ethical guidelines of the 1975 Declaration of Helsinki and was performed according to the recommendations of the Ethics Committee of the Fondazione IRCCS Ca’ Granda. Written informed consent was obtained from each patient. Cohort characteristics are shown in Table 1 and Supplementary Material, Table S1. Serum samples were collected at the time of liver biopsy in all patients after overnight fasting; aliquots were stores and were maintained at −80°C until the analytical procedure. Plasma RBP4 levels were assessed by enzyme-linked immunosorbent assay (Abcam) according to manufacturer's instruction. Non-alcoholic steatohepatitis has been defined as presence of steatosis, ballooning and lobular inflammation (36). Type 2 diabetes and impaired fasting glucose have been defined according to the American Diabetes Association criteria (37).

### Statistics

Data from *in vitro* and *ex vivo* experiments were analyzed using two-tailed Student's *t*-test. *P*-values of <0.05 were considered significant and indicated as * in figures. Bar graphs in figures show mean ± SD of three experiments unless differently specified. Differences in RBP4 levels across PNPLA3 I148M genotypes in humans were analyzed using linear regression analysis or general linear model under a recessive model and after adjusting for confounding factors. *Post hoc* analyses to compare the differences in RBP4 levels between paired genotypes was carried out by Fisher's least significant difference test. Values have been log transformed before entering the model if not normally distributed. Statistical analyses were carried out in IBM SPSS 19.0 for Windows (SPSS, Chicago, IL, USA).

## SUPPLEMENTARY MATERIAL

Supplementary Material is available at *HMG* online.

## FUNDING

This work was supported by the Swedish Research Council (K2013-99X-22230-01-4), the Swedish Diabetes Foundation (DIA2012-014), the Swedish Heart Lung Foundation (20120533), the regional agreement on medical training and clinical research (ALF) between Region Västra Götaland and Sahlgrenska University Hospital (ALFGBG-369381), the Ruth and Richard Julin Foundation (2013juli37071), the co-financed grant from the European Commission, the European Social Fund, and Calabria Region (POR CALABRIA FSE 2007/2013 to P.P. and R.M.M.) and the ‘Borsa Mario Coppo AISF’ of the Italian Association for the Study of the Liver (to R.R.). Funding to pay the Open Access publication charges for this article was provided by the Swedish Research Council (K2013-99X-22230-01-4).

## Supplementary Material

Supplementary Data
