# Supplementary material for: PNPLA3 has retinyl-palmitate lipase activity in human hepatic stellate cells
Source: Hum Mol Genet. 2014 Mar 25;23(15):4077–85. doi: 10.1093/hmg/ddu121 (PMC4082369; doi:10.1093/hmg/ddu121)
Supplement: Supplementary Data [file supp_23_15_4077__index.html]

PNPLA3 has retinyl-palmitate lipase activity in human hepatic stellate cells — PNPLA3 has retinyl-palmitate lipase activity in human hepatic stellate cells — PNPLA3 has retinyl-palmitate lipase activity in human hepatic stellate cells — Supplementary Data 

# PNPLA3 has retinyl-palmitate lipase activity in human hepatic stellate cells

## Supplementary Data

Supplementary Data

**Files in this Data Supplement:**

- Supplementary Data - Docx file
- Supplementary Figure 1 - tif file
- Supplementary Figure 2 - tif file
- Supplementary Figure 3 - tif file
- Supplementary Figure 4 - tif file
- Supplementary Figure 5 - tif file
- Supplementary Figure 6 - tif file
- Supplementary Figure 7 - tif file
- Supplementary Figure 8 - tif file
