# Supplementary material for: PNPLA3 has retinyl-palmitate lipase activity in human hepatic stellate cells
Source: Hum Mol Genet. 2014 Mar 25;23(15):4077–85. doi: 10.1093/hmg/ddu121 (PMC4082369; doi:10.1093/hmg/ddu121)
Supplement: Supplementary Data [file supp_ddu121_ddu121supp.docx]

**SUPPLEMENTARY METHODS AND MATERIALS**

**Antibodies**

The following antibodies were used: rabbit anti-PNPLA3 (Sigma-Aldrich), mouse anti-PNPLA2 (Sigma-Aldrich), mouse anti-LPL (Sigma-Aldrich), mouse anti-LIPC (Sigma-Aldrich), rabbit anti-LIPE (Sigma-Aldrich), mouse anti-V5 (Invitrogen), rabbit anti-AKT (Cell Signaling), rabbit anti-phospho (Ser473) AKT (Cell Signaling) and rabbit anti-calnexin (Sigma-Aldrich).

**Real-time PCR assay**

Real time qPCR was used to assess PNPLA3 mRNA expression in 48 human tissues using TissueScan Human Normal cDNA Array (Origene). To assess mRNA expression in cultured cells, total RNA was isolated from primary human stellate cells, primary human hepatocytes, HEPG2 and CACO-2 cells using the RNeasy Mini Kit (Qiagen). First-strand cDNAs were synthesized from 2 μg RNA using a reverse-transcription kit (Applied Biosystems). The mRNA expression levels were determined in cDNA samples using the delta-delta CT method and normalized to β-actin. TaqMan probes and master mix (Life Technologies) were used in a total volume of 20 µl per reaction. Real-time PCR assay was performed in a 7900HT Fast Real-Time PCR System (Life Technologies).

**Immunoblot analysis**

After washing twice in PBS, cells were harvested and incubated on ice in M-PER® Mammalian Protein Extraction Reagent (Pierce, Thermo Fisher Scientific, Rockford, IL) containing complete protease inhibitor cocktail (Roche). Immunoblot analysis was performed according to standard procedures. Bands were visualized by Chemidoc XRS System and Image Lab Software (Biorad).

**Lipid droplet analysis**

The total area of Oil Red O-stained lipid droplets was determined as described previously ([1](#_ENREF_1)). Pictures were obtained using Axio KS 400 Imaging System and AxioVision 4.8 Software (Zeiss) at 100X magnification.

**Transfection with siRNA and vectors**

ON-TARGET plus PNPLA3 siRNA was obtained from Thermo Scientific. Transfection was performed according to the manufacturer’s instructions. Scrambled siRNA was used as control.

Wild type and 148M mutant PNPLA3 cDNA were cloned in aV5-6His tagged pcDNA3.1 vector (Life Technologies) and transiently transfected into cells using Turbofect liposomes (Fermentas). Transfection efficiency was assessed using an anti-V5 antibody. An empty vector was used as a negative control.

**HEPG2/LX-2 co-culture**

HEPG2 and LX-2 cells were plated in the upper and lower chamber, respectively, of a 24 well trans-well plate with 0.4 μm pore membrane. LX-2 in the lower chamber were transfected with wild type or mutant PNPLA3 and 36 hours after transfection HEPG2 in the upper chamber were incubated with 1μCi/ml of [^3^H]-glycerol plus 300 μM palmitic acid and 0,3% albumin. After 8 hours, cells were harvested and radiolabeled triglycerides were isolated by thin layer chromatography and measured by scintillation counting.

**PNPLA3 effect on triglyceride metabolism analysis in LX-2 cells**

LX-2 cells were incubated with 1 μCi/ml of [^14^C]-palmitic acid plus 300 μM palmitic acid and 0,3% albumin transfected with wild type or mutant PNPLA3 and next incubated with 1 μCi/ml of [^14^C]-palmitic acid plus 300 μM palmitic acid and 0,3% albumin. After 48 hours, cells and media were harvested and intracellular triglycerides and released palmitc acid were measured by scintillation counting after thin layer chromatography lipid fractionation.

**Retinyl-palmitate hydrolysis assessment McA-RH 7777 cells**

McA-RH 7777 stably overexpressing the wild type or the mutant PNPLA3 were harvested and broken in non-denaturing lysis buffer (phosphate buffer saline ph 7.4 plus 1% triton) and cell lysates were incubated 15 minutes at 37 degrees with retinyl-[^14^C]-palmitate. Released [^14^C]-palmitic acid was next measured by scintillation counting. An empty vector was used a s a control.

1 Nicoletti, A., Kaveri, S., Caligiuri, G., Bariéty, J. and Hansson, G.K. (1998) Immunoglobulin treatment reduces atherosclerosis in apo E knockout mice. *J Clin Invest*, **102**, 910-918.

**SUPPLEMENTARY FIGURE LEGENDS**

**Supplementary figure 1. PNPLA3 expression pattern in human tissues.** Human PNPLA3 mRNA expression was measured using cDNA from 48 different human tissues by quantitative real-time PCR. The tissue with the highest CT value was assigned the value of 1.

**Supplementary figure 2.** **PNPLA2, LIPE, LIPC and LPL proteins show low or no expression in primary human hepatic stellate cells.** Immunoblot showing PNPLA3, PNPLA2, LIPE, LIPC and LPL protein amount in primary hepatic stellate cells and human hepatocytes. Blots were obtained from a single PAGE run.

**Supplementary figure 3. PNPLA3 is upregulated by insulin in primary hepatic stellate cells.** Immunoblot showing PNPLA3 protein amount in primary hepatic stellate cells before and after incubation in medium containing insulin (1 nM) for 24 hours. Calnexin was used as loading control. Insulin signaling activation was confirmed by cell lysate immunoblot with anti-phospho-AKT (Ser473) and anti-AKT antibodies at time 0 (-) and after 10 min (+) of insulin treatment.

**Supplementary figure 4. Insulin-mediated PNPLA3 upregulation reduces lipid droplet accumulation in LX-2 cells.** (**A**) Lipid droplet content visualized by Oil Red O (ORO) staining in LX-2 cells incubated with or without retinol (10 µM) and palmitic acid (300 µM) for 48 hours, transfected with PNPLA3 siRNA or control siRNA for 12 hours and incubated with or without insulin (1 nM) for a further 48 hours. **(B)** The ORO-stained area was quantified by BioPix. Data are mean ± s.d. of 3 experiments. **P* < 0.05. **(C)** Immunoblot showing PNPLA3 and PNPLA2 expression in LX-2 cells under conditions described in **A**. Calnexin was used as a loading control. Scale bars, 10 µm.

**Supplementary figure 5. Overexpression of wild type and mutant PNPLA3 does not affect intracellular triglyceride content in LX-2 cells. (A)** Intracellular [^14^C]-tripalmitin after incubation with radiolabeled palmitic acid and subsequent overespression of wild type and mutant PNPLA3. Empty vector (EV) was used as negative control. **(B)** Immunoblot showing transfection efficiency.

**Supplementary figure 6.** **Overexpression of wild type but not mutant PNPLA3 reduces lipid droplet content in LX-2 cells.** (**A**) Lipid droplets visualized by ORO-staining in LX-2 overexpressing V5-tagged 148I or 148M PNPLA3 and incubated with retinol-palmitic acid for 48 hours. Empty vector (EV) was used as negative control. (**B**) ORO-stained area quantified by BioPix. (**C**) Immunoblot showing transfection efficiency.

**Supplementary figure 7. Overexpression of wild type and mutant PNPLA3 in LX-2 cells does not affect intracellular lipid content of HEPG2. (A)** Intracellular ^3^H-tripalmitin in HEPG2 incubated with ^3^H-glycerol and palmitic acid after co-culture with LX-2 overexpressing the wild type or mutant PNPLA3. An empty vector (EV) was used as a control. **(B)** Immunoblot showing transfection efficiency in LX-2 cells.

**Supplementary figure 8 Stable overexpression of wild type and mutant PNPLA3 does not affect retiny-palmitate esterase activity in McA-RH 7777 cells.** (A) [^14^C]-palmitate production after incubation of retinyl-[^14^C]-palmitate with non-denatured cell lysates from McA-RH 7777 stably overexpressing the wild type or the mutant PNPLA3 for 15 min. An empty vector (EV) was used as a control. **(B)** Immunoblot showing stable transfection efficiency in McA-RH 7777 cells.

**Supplementary table 1**

|  | **PNPLA3 I148M genotypes** | | |  |  |  |
| --- | --- | --- | --- | --- | --- | --- |
| **Cohort characteristic** | **II** | **IM** | **MM** | **P*add*** | **P*rec*** | **P*dom*** |
| N | 58 | 65 | 23 | - | - | - |
| Men (%) | 76 | 82 | 74 | 0.652 | 0.598 | 0.599 |
| Age (years) | 47±13 | 51±12 | 47±12 | 0.500 | 0.531 | 0.148 |
| BMI (Kg/m^2^) | 26.7±4 | 27.0±3 | 25.6±3 | 0.084 | 0.146 | 0.156 |
| Glucose (mg/dl) | 95±20 | 101±34 | 97±16 | 0.886 | 0.699 | 0.624 |
| RBP4 (μg/ml) **§** | 50.9±11 | 52.6±14 | 45.6±17 | 0.040 | 0.009 | 0.303 |
| NASH (%) | 42 | 44 | 56 | 0.604 | 0.325 | 0.600 |
| Diabetes or IFG (%) | 26 | 43 | 36 | 0.135 | 0.899 | 0.055 |

Data were analyzed using linear regression analysis after adjusting for confounding factors (age, gender, BMI, NASH, Diabetes or IFG). Values have been log-transformed before entering the model if not normally distributed.

**§** LSD Post hoc test: P value= 0.782 II compared to IM; P value=0.023 II compared to MM

P value=0.012 IM compared to MM

Abbreviations: PNPLA3, patatin-like phospholipase domain-containing protein 3; P*add*, P value under an additive model; P*rec*, P value under a recessive model; P*dom,* P value under a dominant model; II, homozygous for the PNPLA3 148I allele; IM, heterozygous; MM, homozygous for the PNPLA3 148M allele; N, number; BMI, body mass index; RBP4, retinol binding protein 4; NASH, non-alcoholic steato-hepatitis; IFG, impaired fasting glucose; LSD, Fisher's least significant difference.
