# Supplementary figures and images for: PNPLA3 has retinyl-palmitate lipase activity in human hepatic stellate cells
Source: Hum Mol Genet. 2014 Mar 25;23(15):4077–85. doi: 10.1093/hmg/ddu121 (PMC4082369; doi:10.1093/hmg/ddu121)

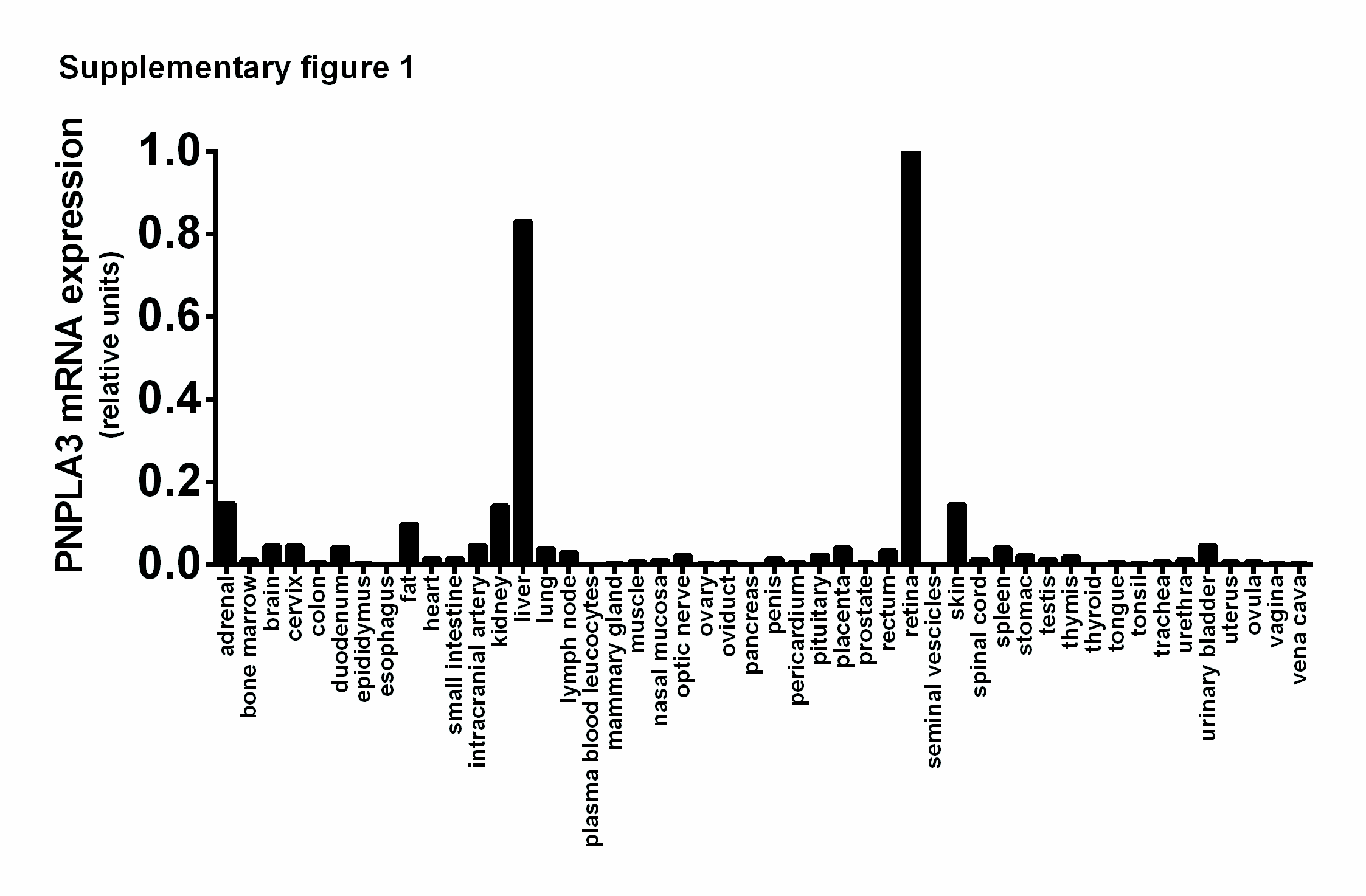

Supplement: Supplementary Data [file supp_ddu121_ddu121supp_fig1.tif]

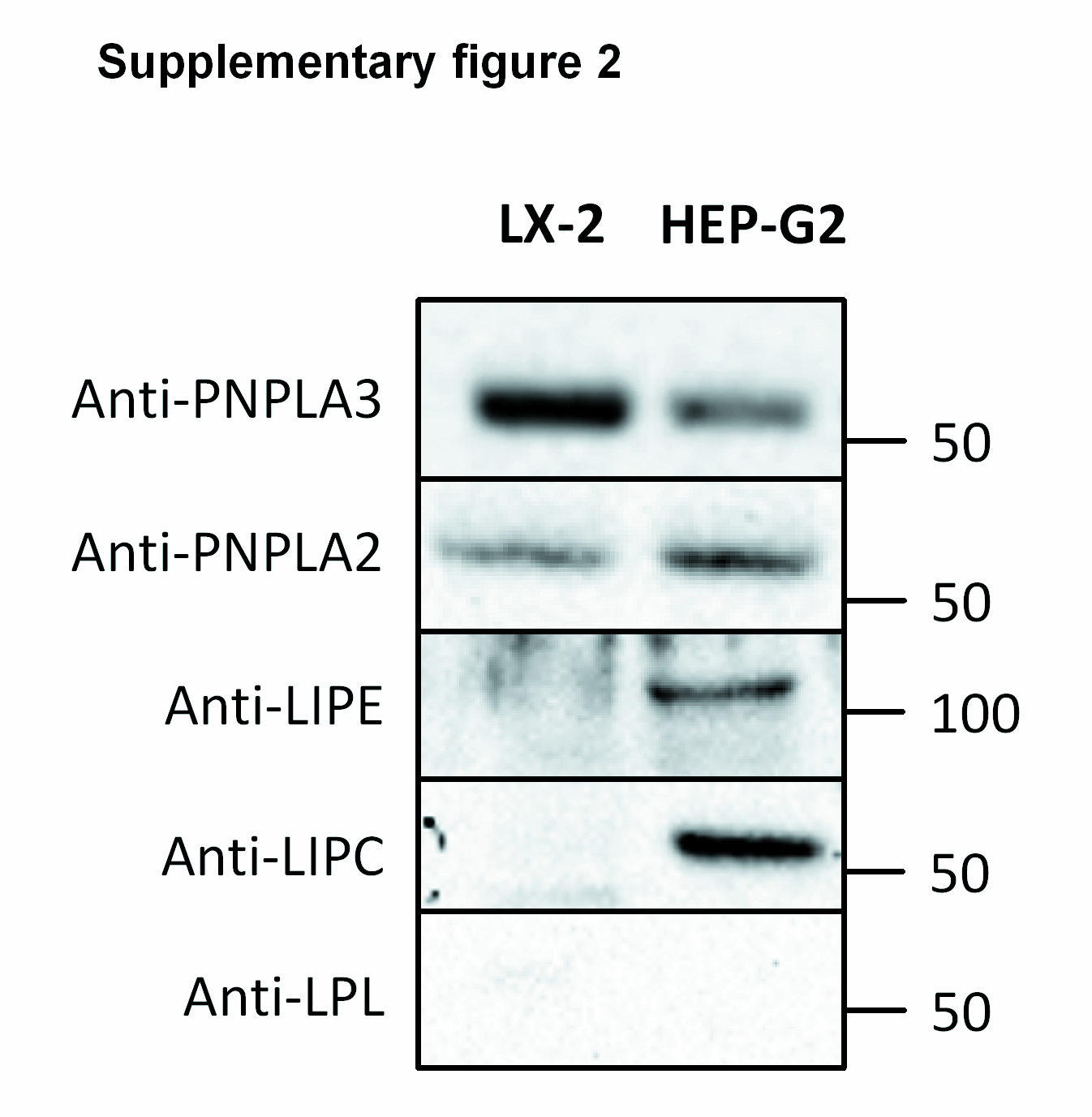

Supplement: Supplementary Data [file supp_ddu121_ddu121supp_fig2.tif]

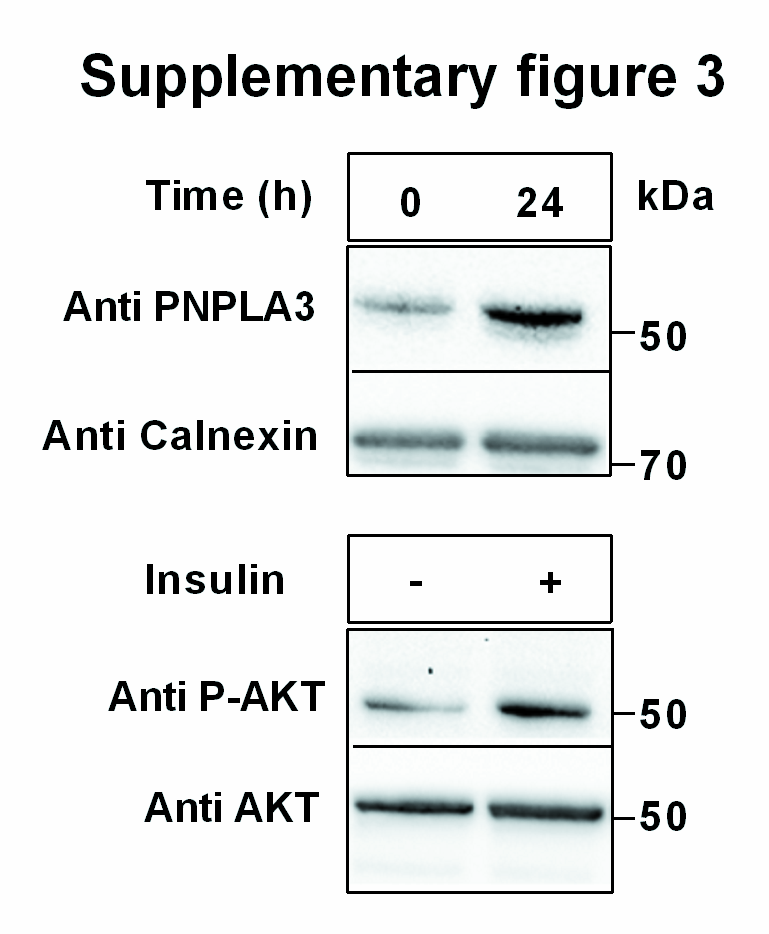

Supplement: Supplementary Data [file supp_ddu121_ddu121supp_fig3.tif]

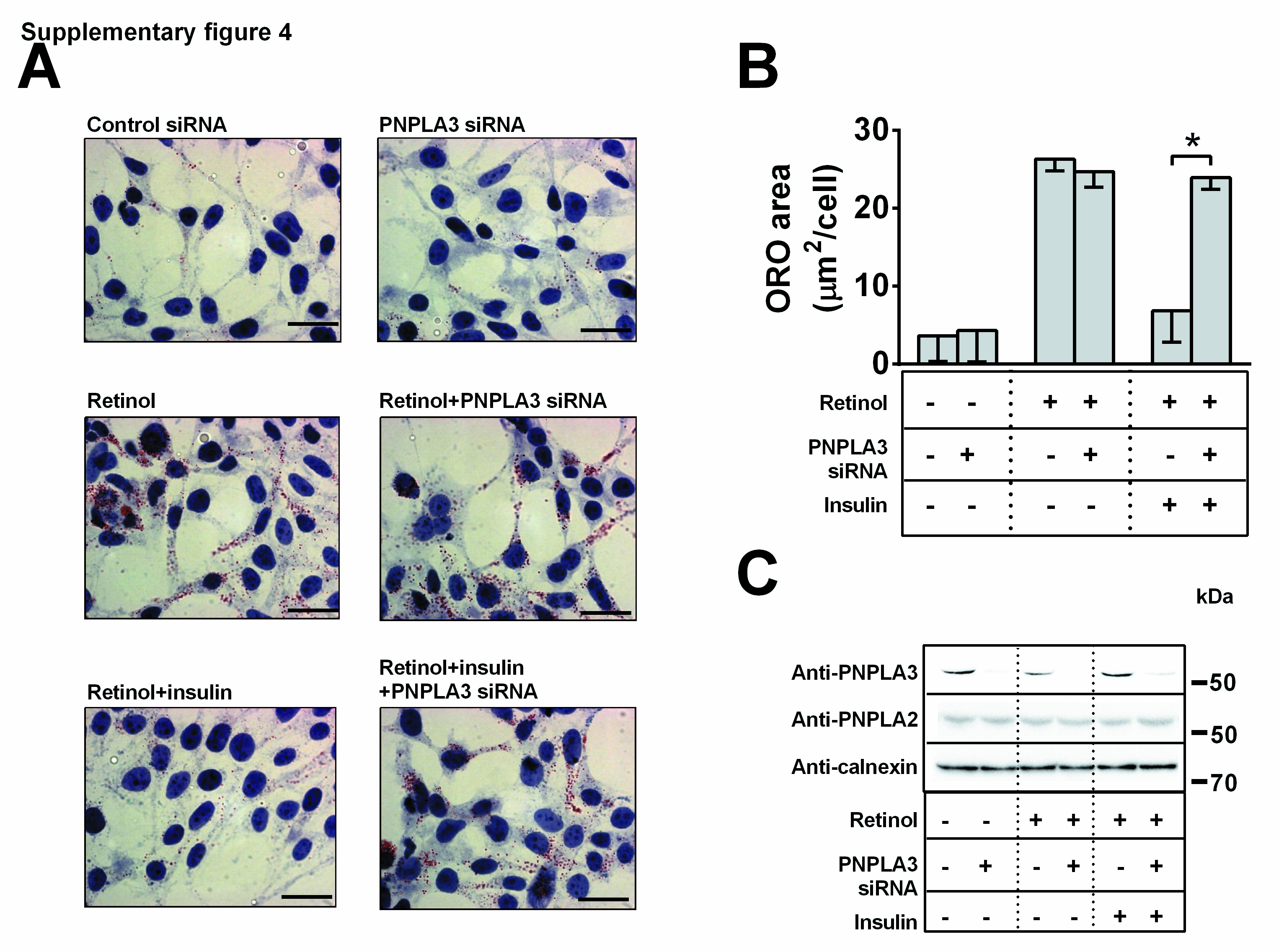

Supplement: Supplementary Data [file supp_ddu121_ddu121supp_fig4.tif]

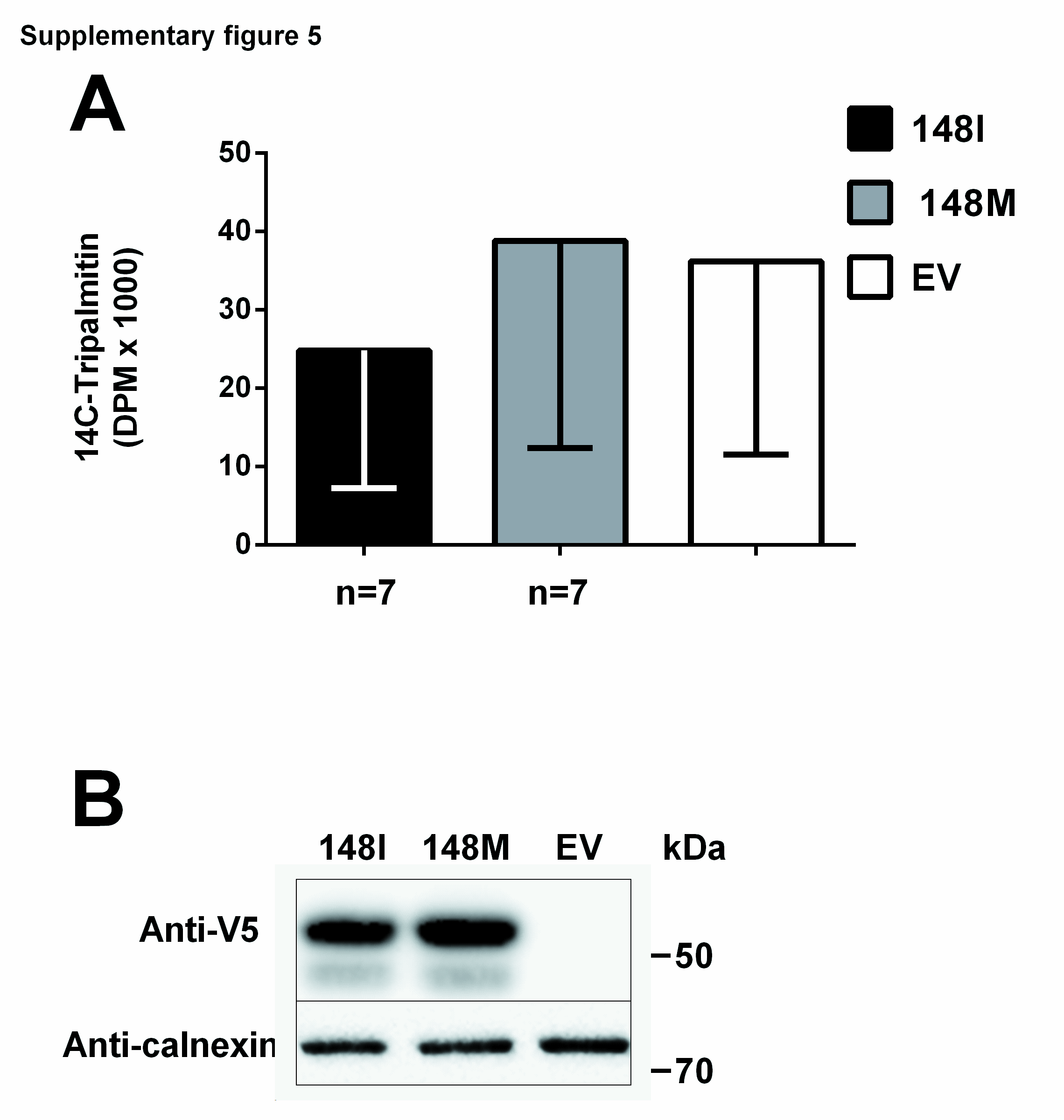

Supplement: Supplementary Data [file supp_ddu121_ddu121supp_fig5.tif]

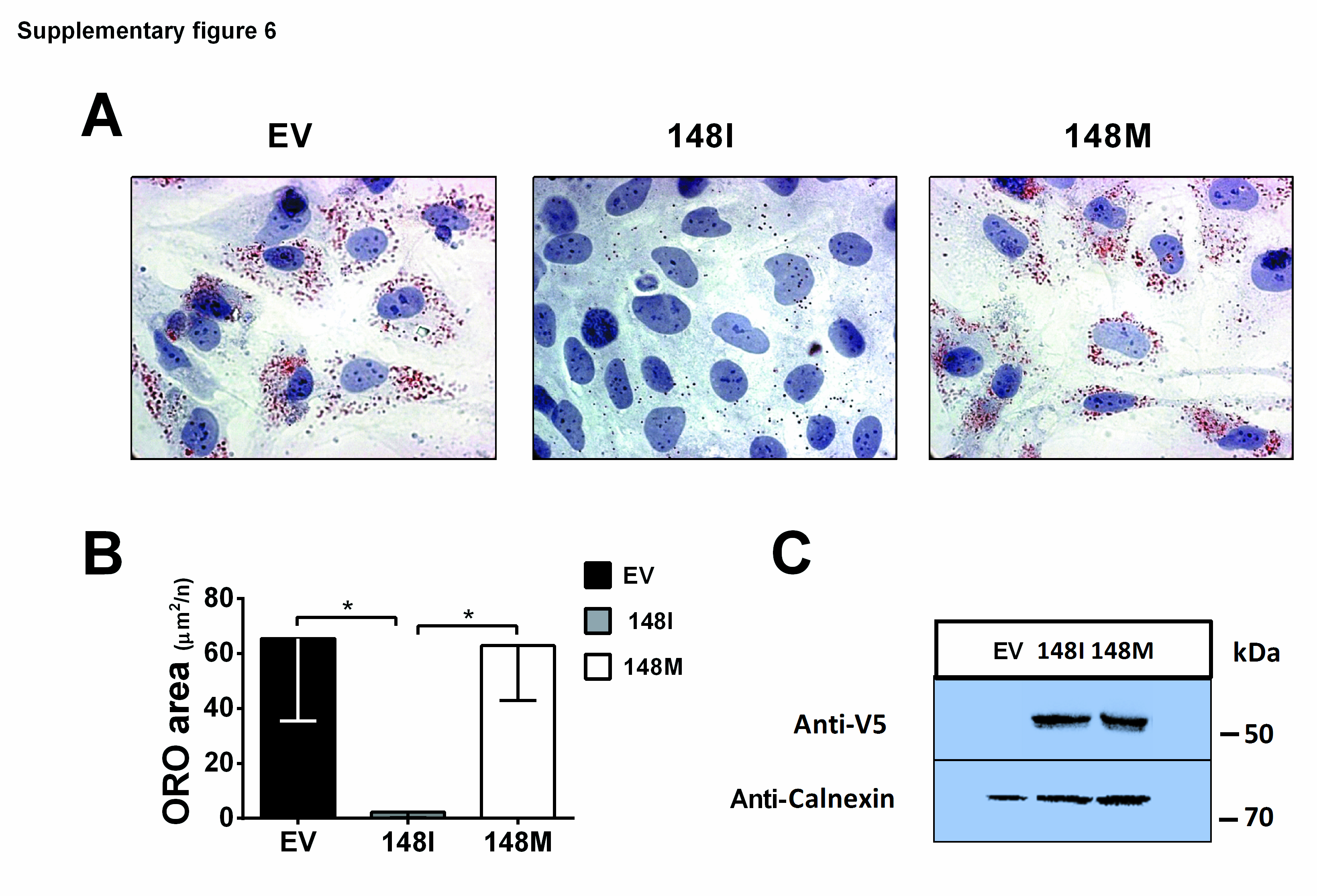

Supplement: Supplementary Data [file supp_ddu121_ddu121supp_fig6.tif]

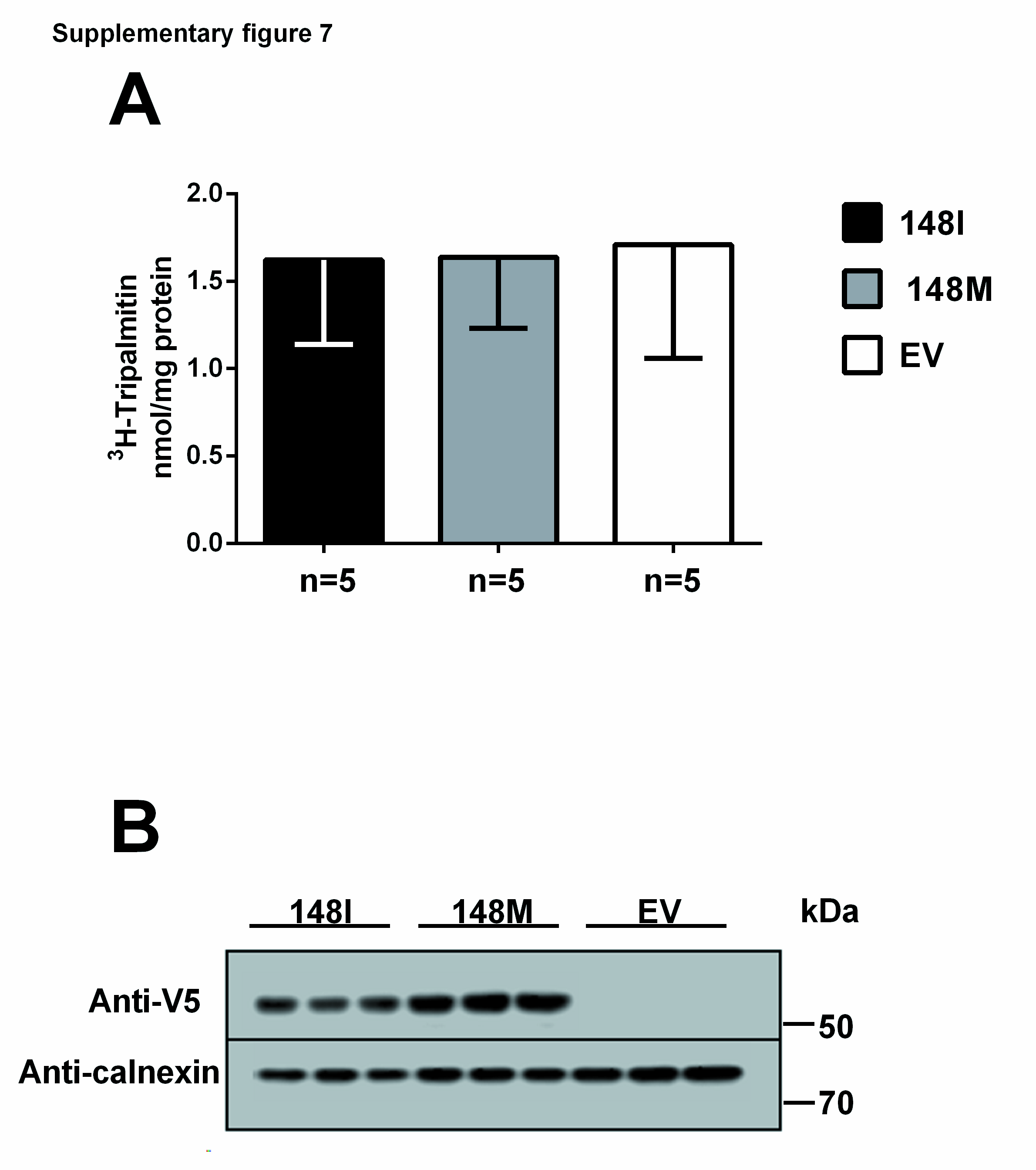

Supplement: Supplementary Data [file supp_ddu121_ddu121supp_fig7.tif]

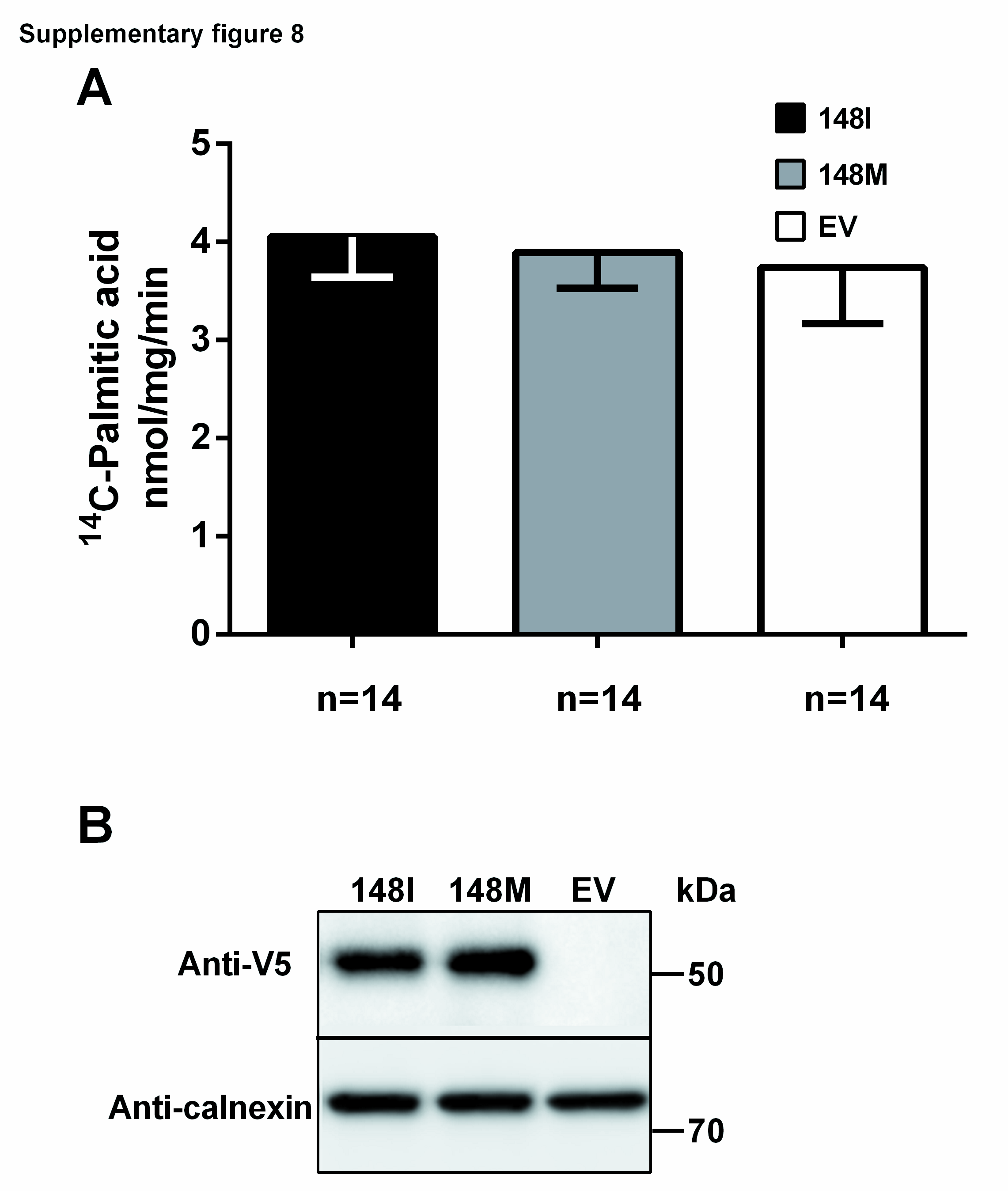

Supplement: Supplementary Data [file supp_ddu121_ddu121supp_fig8.tif]
